# The Mu strain: the last but not least circulating ‘variant of interest’ potentially affecting the COVID-19 pandemic

**DOI:** 10.2217/fvl-2021-0269

**Published:** 2021-11-04

**Authors:** Farid Rahimi, Negin Kamali, Amin Talebi Bezmin Abadi

**Affiliations:** ^1^Research School of Biology, The Australian National University, Canberra, Australia; ^2^Department of Bacteriology, Faculty of Medical Sciences, Tarbiat Modares University, Tehran, Iran

**Keywords:** B.1.621.1, COVID-19, Mu variant, pandemic, SARS-CoV-2, variant of interest

Coronaviruses are genetically variable, enveloped, ssRNA viruses that cause respiratory infections in birds and many mammals, including humans. In the last two decades, the members of coronaviruses have caused three major global disease outbreaks – in 2002, 2012 and 2019 [1]. The scale and consequences of the 2019 pandemic by SARS-CoV-2, causing COVID-19, have been worse than the previous outbreaks by genetically related viruses which caused the Middle East respiratory syndrome (by MERS-CoV) and severe acute respiratory syndrome (by SARS-CoV) [1–3]. By late September 2021, almost 230 million people were reportedly infected with SARS-CoV-2 [4] (while some authors claim that the realistic statistics are underestimates [5,6]). Besides psychological, educational, societal, political, environmental and economic impacts of the pandemic [7–13], additional justifiable concerns have grown about circulation of the new SARS-CoV-2 variants in different countries. Emergence of the variants indicate that genetic and phenotypic alterations of SARS-CoV-2 have begun – well within a year of the pandemic.

Viral alterations ensue recombination and insertion or deletion and misincorporation of nucleotides in the sequence of the viral genome. These are driven by natural selection of the viral traits that collectively promote survival and propagation, including virulence, efficient transmission and immune evasion [14]. Increases in the COVID-19 cases in different countries by mid-September 2021 can be explained by the emergence of many SARS-CoV-2 variants, increasingly confirmed in clinical samples by genome sequencing. Reportedly since May 2021, the Delta (B.1.617.2) SARS-CoV-2 variant [15,16] heavily dominated the pandemic picture in five continents, causing alarming numbers of daily fatalities.

The WHO designated a recent SARS-CoV-2 variant, the Mu variant [17], as a variant of interest (VOI) on 30 August 2021 while it was first reported in Colombia in January 2021 [18]. The Mu variant is classified as B.1.621 (PANGO lineage) or 21H (nextstrain clade). The WHO requirement for ‘comparative assessment of virus characteristics’ of a new SARS-CoV-2 variant should increase our understanding and awareness of this VOI. The number of countries with reports of this VOI is rising. This VOI was detected in more than 51 countries according to the GISAID initiative updated on 18 September 2021 [19,20]. Some experts and media reports argue that the Mu VOI may escape immunity induced by vaccines or past infections [21].

Several mutation clusters detected in its sequence may enable this VOI to evade the human immune responses due to vaccination or past infections. Immune evasion may challenge the effectiveness of the present vaccines used to fight the pandemic [18]. To date, nine common mutations were found in the spike protein of the Mu (B.1.621) variant: T95I (in 93.6% of sequences), Y144S (77.1%), Y145N (83.9%), R346K (96.1%), E484K (93.9%), N501Y (94.3%), D614G (97.3%), P681H (97%) and D950N (89.3%) [20,22]. Deletion of 144/145 was seen in 0.6% of all sequences [20]. The most effective mutation sites occur at the receptor-binding domain of the virus spike protein which mediates attachment and entry of the virus into the host cells. Mutations in the receptor-binding domain of the spike protein are thought to increase transmissibility in the Alpha (B.1.1.7), Beta (B.1.351), Gamma (P.1) and A.23.1 variants; increase virulence in Alpha (B.1.1.7); and reduce vaccine effectiveness against Alpha (B.1.1.7), Beta (B.1.351) and A.23.1 strains [14]. Many of the mutations that the Mu variant carries have already been detected among the previous variants of concern (VOCs) with >75% prevalence in at least one lineage. For example, some common mutations in the spike protein (E484K, N501Y, D614G) are shared between the Mu and other VOIs or VOCs (according to the website of the European Centre for Disease Prevention and Control [23]). T95I has been confirmed in 35.2% of the Delta (B.1.617.2); E484K and N501Y were detected with high frequency in Gamma (P.1) and Beta (B.1.351); N501 in Alpha (B.1.1.7); and D641G in Alpha (B.1.1.7), Beta (B.1.351), Delta (B.1.617.2) and Gamma (P.1) VOCs [20,22]. The Mu variant shares many other mutations with the presently classified VOIs and VOCs (details of the mutations carried by the SARS-CoV-2 variants are available at https://covariants.org/shared-mutations). Such common mutations may enable the Mu lineages to efficiently enter the host cells, evade the immune system or challenge the effectiveness of the presented vaccines. Thus, assessments of the risks associated with the Mu variant may change.

## Circulating VOIs & VOCs are & will be an ongoing challenge during the pandemic

According to WHO, a SARS-CoV-2 lineage qualifies as a VOI if the genetic changes can affect or predictably affect the viral characteristics such as disease severity (virulence); immune, diagnostics or therapeutics evasion; or transmissibility; and cause significant community transmission or multiple clusters in many countries [17]. The VOC qualification; however, includes high transmissibility or detrimental changes in the COVID-19 epidemiology; or high virulence or altered clinical presentations; or a decreased effectiveness of public-health countermeasures or available diagnostics, vaccines or therapeutics [17]. Indeed, VOCs are qualified by one or many of the above characteristics with severe clinical consequences globally. In late August, the Mu VOI was prevailing among 39% of all infected Columbian cases [24]; this proportion may become higher in other countries. A Japanese group has raised major concerns regarding circulation of the vaccine-resistant SARS-CoV-2 variants [25], reporting that the Mu lineages are significantly (p = 0.031) resistant to convalescent-serum-mediated neutralization compared with other VOIs or VOCs presently classified [25]. This report has detrimental implications for the naive and fully vaccinated individuals globally if they abandon the public-health countermeasures, including the wearing of face masks, physical distancing and respiratory or manual hygiene. Emergence of the variants also reaffirms the importance of maintaining high-quality ventilation systems inside office towers, high-rise residences, airplanes, office spaces, quarantine facilities and classrooms or lecture rooms. The continuing challenge of the pandemic once more emphasizes the importance of acknowledging the learnt new facts and discarding misinformation about SARS-CoV-2 and its variants [26].

Besides the Mu (B.1.621) variant, four other VOIs have emerged and partially circulated globally. These include Lambda (C.37), Kappa (B.1.617.1), Iota (B.1.526) and Eta (B.1.525) variants. Realistically, the list of VOIs or VOCs involved in the COVID-19 pandemic is not complete yet. Though qualifying the Mu VOI as a VOC may be premature, studying or recording this VOI’s (and other VOIs’) characteristics including interpersonal transmissibility, virulence, resistance to vaccination and evasion of immunity due to past infections will be valuable for guiding future countermeasures, such as vaccine formulations, during the pandemic. This challenges our present aim of achieving herd immunity by vaccination or past infections. Thus contextually, we postulate that herd immunity may be required realistically against every new SARS-CoV-2 variant that begins or tends to dominate the pandemic statistics in some countries or globally. Thus, opportune and firm consolidated surveillance systems should be implemented globally to monitor and characterize the variants and examine their effects on the present and rapidly administered mass vaccination campaigns globally. This requirement; however, may be challenging in countries that lack capabilities, funding or the appropriate infrastructure for genome sequencing.

## Future perspective

According to the WHO assessments, the Mu variant is less clinically problematic than the other SARS-CoV-2 variants, including Alpha (B.1.1.7) or Gamma (P.1). Besides Mu, four other VOIs, including Lambda (C.37), Kappa (B.1.617.1), Iota (B.1.526) and Eta (B.1.525), have emerged and propagated. Nonetheless, understanding the exact biological features of the Mu strain is necessary to ensure that healthcare systems are not overwhelmed by another high rate of hospitalizations due to a new, potentially deadly variant. Although considering the Mu variant as a VOC may be premature now, its dominant circulation and high infection rates in Colombia – as the ‘Mu epicenter’ – justifies characterizations of this and other variants by validated mutation-detecting nucleic acid amplification and confirmatory genomic sequencing to corroborate the viral transmission, pathogenesis, epidemiology, clinical features and potential to resist or evade the human immune responses or vaccinations.

The collective understanding of, or even doubts about, the new variants can only emphasize that accepting to live with the virus as a ‘new normalcy’ must not equate to complacence or abandonment of the public-health countermeasures. This may be challenging in societies that resist wearing masks or with individuals that become complacent or wear inappropriate facemasks. The same challenges are graver in poor countries that strive economically to achieve proper healthcare resources and maintain the countermeasures. Furthermore, accepting and propagating the facts instead of misinformation about SARS-CoV-2, COVID-19 and the pandemic is as fundamental now as it was at the beginning of the pandemic [26].
